# Supplementary material for: Compartmentation of cGMP Signaling in Induced Pluripotent Stem Cell Derived Cardiomyocytes during Prolonged Culture
Source: Cells. 2022 Oct 17;11(20):3257. doi: 10.3390/cells11203257 (PMC9600086; doi:10.3390/cells11203257)
Supplement: Supplementary file 1 [file cells-11-03257-s001.zip › cells-1917349-supplementary materials.pdf]

Table S1. Primers used on cDNA IMR90 iPS-CMs aged day 30 and day 90.

mRNA	Primer Sequence
Phosphodiesterase 1A (PDE1A)	*Fwd:* TTGGCTTCTACCTTTACACGGA<br>*Rev:* AGGGCAAATACATCGAAAGACC
Phosphodiesterase 1B (PDE1B)	*Fwd:* ATGAGACACGGCAAATCTTGG<br>*Rev:* TGCACAATGCTTCGGAACTTG
Phosphodiesterase 1C (PDE1C)	*Fwd:* GATGTGGACAAGTGGTCCTTTG<br>*Rev:* GGGGATCTTGAAACGGCTGA
Phosphodiesterase 2A (PDE2A)	*Fwd:* CCTCCTGTGACCTCTCTGAC-C<br>*Rev:* TGAACTTGTGGGACACCTT-GG
Phosphodiesterase 3A (PDE3A)	*Fwd:* TCACAGGGCCTTAACTTTAC-AC<br>*Rev:* GGAGCAAGAATTGGTTTGT-CC
Phosphodiesterase 3B (PDE3B)	*Fwd:* CCTCAGGCAGTTTATACA-ATG<br>*Rev:* TGCTTCTTCATCTCCCTGCT-C
Natriuretic Peptide Receptor 1 (NPR1)	*Fwd:* CTTCGGTGTCAAGGACGAGTA<br>*Rev:* GGTAGGCGTAGAGCATGAGC
Natriuretic Peptide Receptor 2 (NPR2)	*Fwd:* TGACCCCGACCTGCTGTTA<br>*Rev:* CGAACCAGGGTACGATAATGG
β_3- adrenergic receptor (ADRB3)	*Fwd:* GACCAACGTGTTCGTGACTTC<br>*Rev:* GCACAGGGTTTCGATGCTG
Human glyceraldehyde 3-phosphate dehydrogenase (hGAPDH)	*Fwd:* AATCCCATCACCATCTTCCA<br>*Rev:* TGGACTCCACGACGTACTCA

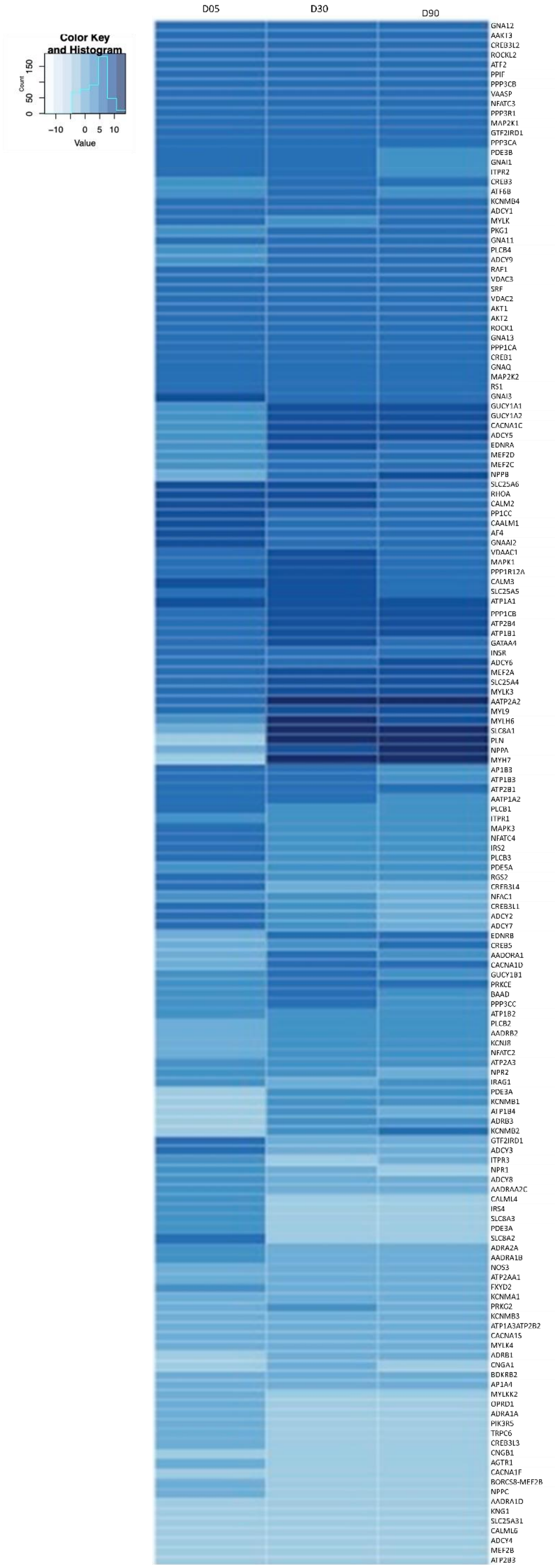

Figure S1. Heatmap of gene expression of genes involved in the cGMP-PKG pathway in induced pluripotent stem cell cardiomyocytes (iPS-CM) as they were cultured for 5, 30 and 90 days.

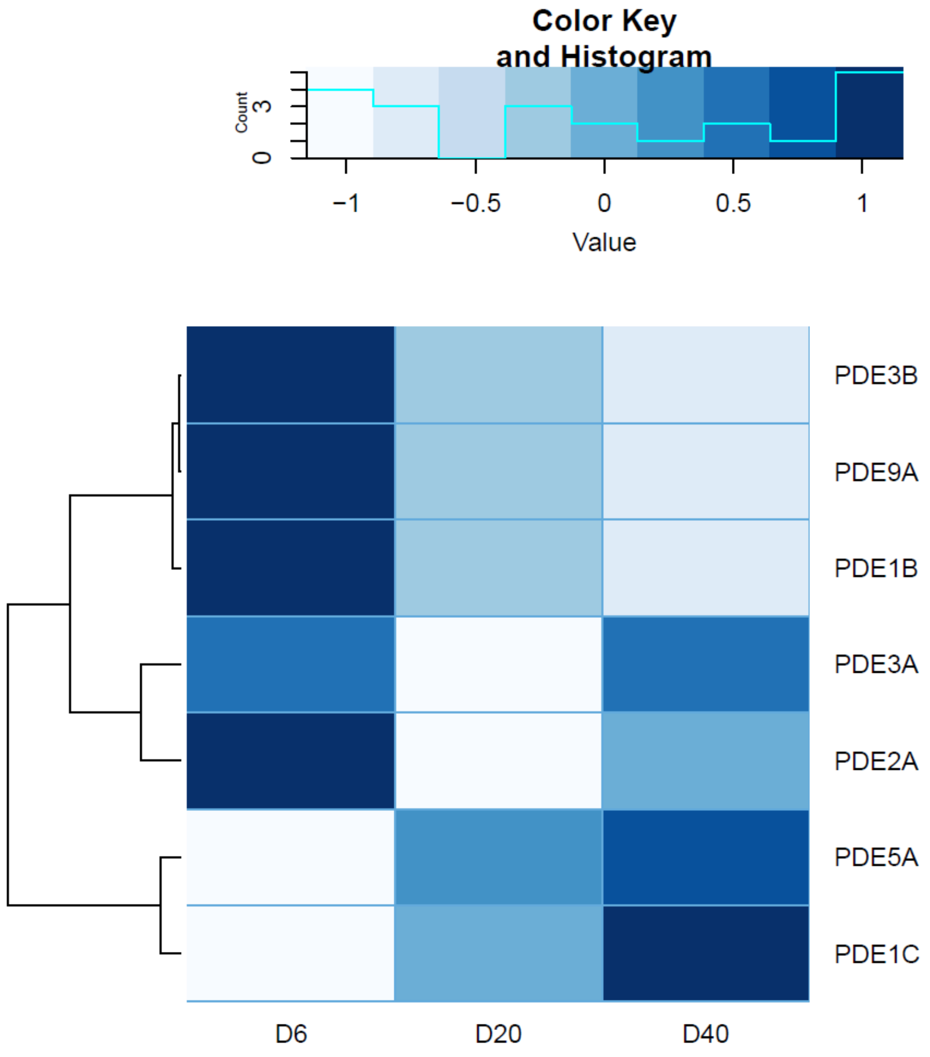

Figure S2. Expression of various PDEs in H9 hiPS-CMs as revealed by RNA-seq.

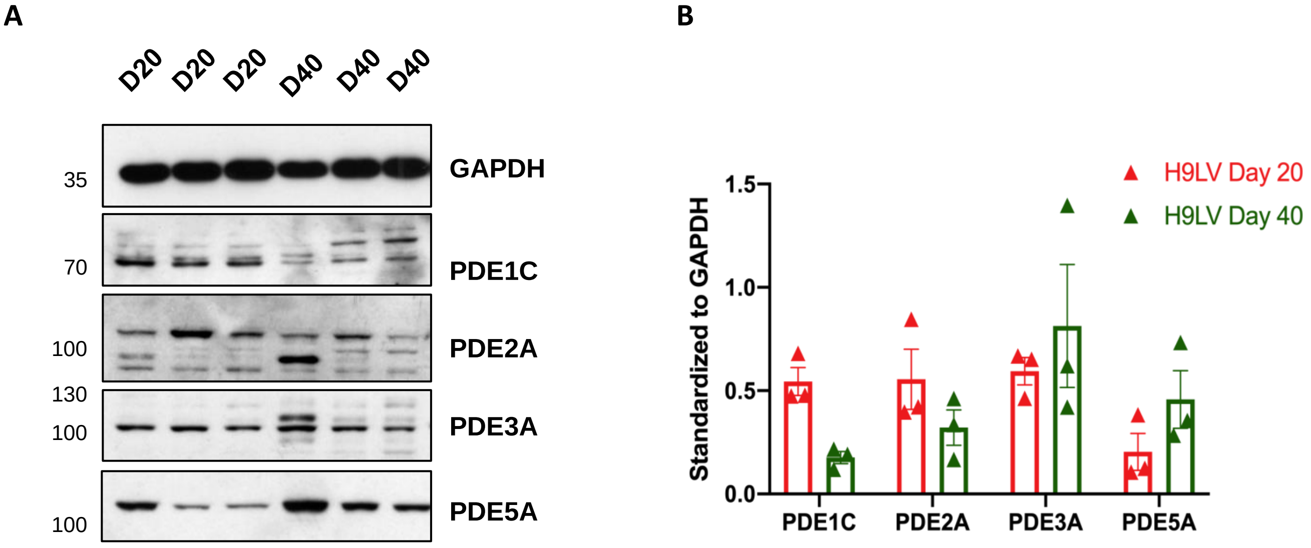

Figure S3. Expression of various PDEs in H9 hiPS-CMs at early and late stage in culture. A. Western blot of PDEs at Day 20 and Day 40 in culture; B. Quantification band density of blots in A.

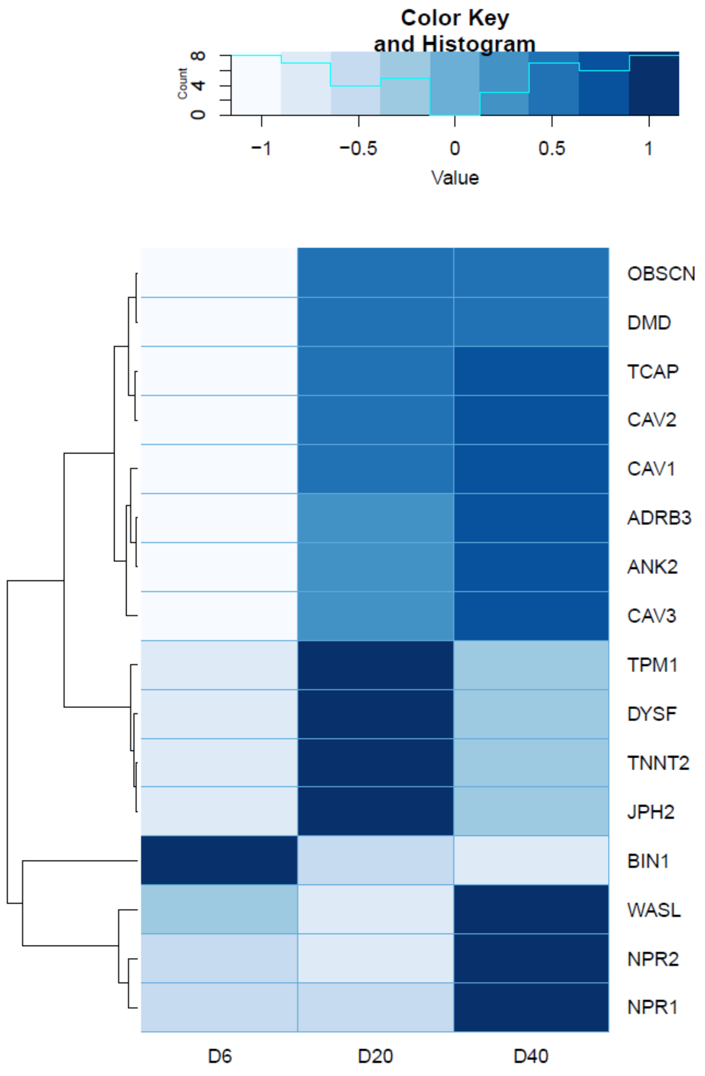

Figure S4. Expression of genes relevant to myocyte maturation in H9 iPS-CMs as revealed by RNA-seq.

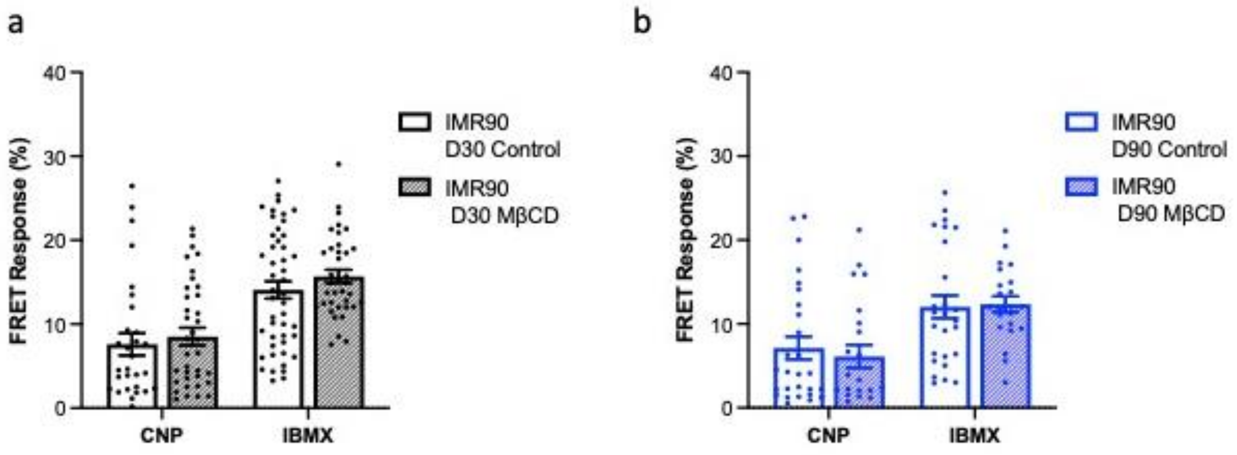

Figure S5. cGMP levels in control and caveolae depleted (MβCD) IMR90 iPS-CMs aged (a) day 30 and (b) day 90 upon C-type peptide (CNP) stimulation and general phosphodiesterase inhibition (IBMX).
